# Editorial: Mechanism and pharmacodynamic material basis of neurodegenerative disease therapies

**DOI:** 10.3389/fnins.2023.1254881

**Published:** 2023-07-21

**Authors:** Erxi Wu, Ronghu Ke, Dan Qi, Jason H. Huang

**Affiliations:** ^1^Department of Neurosurgery and Neuroscience Institute, Baylor Scott and White Health, Temple, TX, United States; ^2^Department of Medical Education, Texas A&M University School of Medicine, Temple, TX, United States; ^3^Department of Pharmaceutical Science, Texas A&M University School of Pharmacy, College Station, TX, United States; ^4^Department of Oncology, Livestrong Cancer Institutes, Dell Medical School, The University of Texas at Austin, Austin, TX, United States; ^5^Department of Neurosurgery, Baylor College of Medicine, Temple, TX, United States

**Keywords:** neurodegenerative disease, Alzheimer's disease, Parkinson's disease, therapies, natural compounds, metabolism, multiomics

Neurodegenerative disorders exert a profound global impact, causing a gradual deterioration of the nervous system and leading to the progressive loss of neurons. These conditions disrupt vital communication pathways, resulting in impaired cognition, memory, behavior, sensory perception, and motor function (Wilson et al., 2023). The high prevalence of neurodegenerative diseases, such as Alzheimer's disease (AD) and Parkinson's disease (PD), among the aging population underscores the urgent need for effective treatments. Currently, available treatments for neurodegeneration are limited or non-existent, imposing significant socioeconomic and personal burdens.

In recent years, the emergence of “omic” approaches, such as genomics, transcriptomics, epigenomics, and metabolomics, has provided unprecedented insights into the understanding of neurodegenerative diseases (Badhwar et al., 2020). Despite these advancements, therapeutic interventions capable of curing these conditions are still lacking. Therefore, it is imperative to actively pursue new drug candidates and effective treatment strategies. The primary focus of this Research Topic is to utilize “omics” in studying biomarkers, pathogenesis, and disease progression, while also identifying novel therapeutic targets for neurodegenerative diseases and related conditions. This issue accepted 10 exceptional papers, including original research articles and reviews. Through these contributions, we aim to shed light on the latest advancements and discoveries in the field, fostering a deeper understanding of neurodegenerative diseases and providing a platform for the development of novel treatment modalities.

## Exploring potential therapeutic natural compounds for neurodegenerative diseases such as AD

Currently, effective therapies for neurodegenerative diseases such as AD remain elusive. AD is the most prevalent neurodegenerative disorder and the leading cause of dementia among the elderly (Alzheimer's Association, 2023). Clinical treatment of AD focuses primarily on syndrome control and lacks effective therapy. Although the recent approval of two drugs, aducanumab and lecanemab, by the United States FDA for treating AD has sparked controversy, there is growing interest in exploring natural compounds as a promising avenue for AD treatment (Zhang et al., 2012; Xia et al., 2017). An important aspect of this issue is the significant number of studies dedicated to investigating novel approaches for the treatment of AD, with a particular emphasis on utilizing natural compounds. These studies delve into the exploration of alternative therapeutic strategies, aiming to identify potential interventions that harness the power of nature. By dedicating considerable attention to this aspect, this issue contributes to the growing body of research dedicated to advancing AD treatment through the exploration of natural compounds.

Zhou et al. investigated the therapeutic effects of Alpiniae oxyphyllae Fructus (AOF) on AD using metabolomics. They found that treatment with AOF in APP/PS1 transgenic mice with AD reversed significant alterations in 31 metabolites in plasma, including notable changes in bile acids, indicating the potential of AOF to ameliorate AD symptoms by modulating bile acid metabolism and highlighting the need for further research into its therapeutic mechanisms.

Liu et al. found that Ginkgo biloba L. leaf extract (GBLE) reversed the significant alterations in 60 metabolites observed in APP/PS1 mice with AD, similar to the response seen with donepezil, an established AD medication. This suggests that GBLE restores lipid metabolic balance and may contribute to its neuroprotective effects in AD treatment, providing insights into AD pathogenesis and the therapeutic potential of GBLE.

Li et al. demonstrated that total saikosaponins (TS) from Radix bupleuri (Chaihu) improved cognitive function, reduced amyloid-beta (Aβ) generation and plaque deposition, enhanced autophagy, downregulated β-secretase 1 (BACE1), nuclear factor-κB (NF-κB), and inflammatory factors, activated nuclear factor 2 (Nrf2), and potentially regulated gut microbiota while alleviating oxidative stress, suggesting TS as a potential therapeutic strategy for AD pending further investigation into the underlying mechanisms.

Wang et al. investigated the therapeutic effect and mechanism of Anemarrhenae Rhizoma (AR) on AD using a rat model induced by D-galactose and aluminum chloride (AlCl_3_). Metabolomics analysis using mass spectrometry identified 32 significantly affected metabolites in the serum, indicating the influence of AR on various metabolic pathways. The findings from behavior studies, histopathological observations, and biochemical analyses supported the therapeutic potential of AR in AD prevention and treatment, providing valuable insights into its therapeutic mechanism.

Wu et al. identified phenylpropanoid sucrose esters from Fallopia dentatoalata that exhibited moderate inhibitory effects against acetylcholinesterase (AChE) and potential inhibitory effects against butyrylcholinesterase (BuChE), with certain compounds acting as non-competitive inhibitors of AChE and others acting as competitive inhibitors of BuChE, suggesting their potential as anticholinesterase therapeutics for AD.

Song et al. investigated the protective effects of matrine (MAT) on retinal ganglion cells (RGCs) in optic neuritis (ON), a condition associated with vision loss and inflammation. The study found that MAT treatment reduced inflammation and demyelination, and promoted RGC survival by upregulating the expression of Sirtuin 1 (SIRT1) and its downstream molecules Nrf2 and PGC-1α, suggesting its potential as a therapeutic intervention for optic neuritis.

## Investigating immune infiltration in AD for developing therapeutic targets

Zhang et al. analyzed the immune cell composition in the entorhinal cortex of AD patients and identified 81 immune-related differentially expressed genes. They found decreased lymphocyte scores and increased myeloid cell scores in AD patients and identified 37 genes involved in innate immunity, with eight genes being potential drug targets. These findings provide insights into potential therapeutic targets relevant to the immune components in AD, contributing to the understanding and treatment of the disease.

## Highlighting the roles of exosomes in AD development and diagnosis

The review by Zou et al. offers a comprehensive overview of the roles of exosomes in AD, highlighting their involvement in AD development, their potential as biomarkers in various body fluids, and their significance in AD diagnosis and treatment. This review underscores the potential of exosomes as valuable tools for AD diagnosis, treatment, and clinical management of AD-related dementia.

## Identifying BAG5 as a potential diagnostic biomarker for PD with the PINK1 R492X mutation

In their study, Fu et al. found lower expression levels of Bcl2-associated athanogene 5 (BAG5) in the skin tissues of PD patients with the R492X PINK1 mutation than healthy controls. They also discovered that BAG5 interacts with the R492X mutated PINK1 protein and facilitates its degradation through the ubiquitin/proteasome-dependent pathway. These findings highlight the potential of BAG5 as a diagnostic biomarker and provide insights into therapeutic targets for PD patients with the R492X PINK1 mutation.

## Targeting ferroptosis for the treatment of ischemic stroke

Wei et al.'s review highlights the role of the neurovascular unit (NVU) in ischemic stroke and underscores ferroptosis as a significant factor in stroke progression and NVU regulation, suggesting that targeting ferroptosis holds promise as a therapeutic strategy to prevent severe brain damage and reduce the risk of neurodegenerative conditions associated with ischemic stroke.

## Perspectives

The primary objective of this Research Topic was to provide the scientific community with valuable insights into recent advancements in studying the mechanisms and pharmacodynamic material basis of therapies for neurodegenerative diseases. By doing so, we aimed to contribute to the development of more effective therapeutic strategies, with the ultimate goal of benefiting patients worldwide who are affected by these debilitating conditions. Through this collective effort, we aspire to make significant strides toward improving the lives of individuals suffering from neurodegenerative diseases.

## Author contributions

All authors listed have made a substantial, direct, and intellectual contribution to the work and approved it for publication.
